# The Role of TLR2 and 4-Mediated Inflammatory Pathways in Endothelial Cells Exposed to High Glucose

**DOI:** 10.1371/journal.pone.0108844

**Published:** 2014-10-10

**Authors:** Harshini Mudaliar, Carol Pollock, Jin Ma, Huiling Wu, Steven Chadban, Usha Panchapakesan

**Affiliations:** 1 Renal Research Group, Kolling Institute of Medical Research, University of Sydney, Royal North Shore Hospital, St Leonards, New South Wales, Australia; 2 Renal Medicine Royal Prince Alfred Hospital and Collaborative Transplant Research Group, University of Sydney, Camperdown, New South Wales, Australia; University of Padua, Italy

## Abstract

Postprandial hyperglycemia induces inflammation and endothelial dysfunction resulting in vascular complications in patients with diabetes. Toll-like receptors (TLRs) are central to the regulation of inflammatory responses through activation of nuclear factor-kappa B (NF-ĸB). This study examined the role of TLR2 and 4 in regulating inflammation and endothelial dysfunction when exposed to fluctuating glucose concentrations. HMEC-1 cells (a human microvascular endothelial cell line) were exposed to control (5 mM), 30 mM (high), fluctuating (5/30 mM) and 11.2 mM glucose (approximate glycaemic criteria for the diagnosis of diabetes mellitus) for 72 h. Cells were assessed for TLR2, 4, high mobility group box -1 (HMGB1), NF-ĸB, monocyte chemoattractant protein-1 (MCP-1), interleukin-8 (IL-8), intercellular adhesion molecule-1 (ICAM-1) and vascular cell adhesion molecule-1 (VCAM-1). Fluctuating glucose concentrations maximally upregulated TLR4 but not TLR2 expression with increased NF-ĸB activation, IL-8 and ICAM-1 expression. HMGB1 was increased in the supernatants of cells exposed to 30 mM and 11.2 mM glucose compared to control. The addition of recombinant HMGB1 induced NF-ĸB activation and synthesis of proinflammatory cytokines and chemokines, which were prevented by TLR2 or 4 signalling inhibition. An additive effect when both TLR2 and 4 signalling pathways were inhibited was observed. However, only inhibition of TLR4 signalling suppressed the synthesis of MCP-1, IL-8 and ICAM-1. *In vivo*, streptozotocin-induced diabetic mice exhibited an increase in glomerular ICAM-1 which was not evident in TLR2^-/-^ or TLR4^-/-^ diabetic mice. Collectively, our results suggest that targeting the signalling pathway of TLR2 and 4 may be of therapeutic benefit in attenuating vascular inflammation in diabetic microangiopathy.

## Introduction

Postprandial hyperglycemia is one of the earliest abnormalities of glucose homeostasis observed in patients with type 2 diabetes mellitus and is defined as plasma glucose level exceeding 7.8 mM glucose (140 mg/dl) [1], [2]. Several prospective studies have drawn a strong correlation between postprandial hyperglycemia and the incidence of microvascular and macrovascular complications [3]–[5]. Macrovascular complications in diabetic patients consist mainly of cardiovascular events [6]. However, microvascular complications result in considerable morbidity, notably diabetic nephropathy and retinopathy [3], [5]. A hallmark of diabetic vascular pathology is endothelial dysfunction. It is well established that the association between inflammatory and metabolic abnormalities contribute to the multifactorial pathogenesis of endothelial dysfunction in diabetes.

Toll-like receptors (TLRs) are evolutionarily conserved innate receptors which activate the NF-κB pathway leading to the synthesis of proinflammatory cytokines and chemokines upon the recognition of microbial components or ligands generated at sites of injury [7]–[9]. Therefore, an investigation into the mechanisms underlying the relationship between fluctuating glucose concentrations, endothelial dysfunction and inflammatory signalling pathways regulated by TLRs in diabetic microangiopathy may suggest novel therapeutic targets.

The Diabetes Control and Complications Trial (DCCT), U.K Prospective Diabetes study (UKPDS) and the Kumamato Study [3], [5], [10] have shown that treatment programs implementing tight glycemic control ameliorate the risk of microvascular complications in patients with type 2 diabetes mellitus including diabetic nephropathy and retinopathy. Postprandial glucose fluctuations have also been demonstrated to induce inflammation and the upregulation of adhesion molecules, which may result in endothelial dysfunction [11], [12]. Although several laboratory studies and clinical data support the deleterious effect of oscillating hyperglycemia on endothelial dysfunction compared to chronic sustained hyperglycemia [13]–[15], the impact of variability on the cardiovascular outcomes is unclear. Given that acute increases in glucose may in part contribute to vascular tissue damage, the exact mechanisms by which inflammation is modulated in endothelial dysfunction remains poorly understood.

In patients with type 2 diabetes, endothelial dysfunction is present early in the disease [16] and there is increasing evidence that the human vascular endothelium provides an important site of regulation and amplification of inflammatory responses. Endothelial cells have been shown to be active producers of inflammatory molecules such as chemokines, adhesion molecules and cytokines, all of which are involved in the pathophysiology of diabetic microangiopathy [17].

There is growing evidence that TLRs, especially TLR2 and 4, are involved in the pathogenesis of diabetic microangiopathy [18]–[20]. However, the mode of activation of TLRs in diabetic microangiopathy and their relationship to endothelial dysfunction has not been well characterised. To date, 11 members of the TLR family have been identified in humans [21]. They function as important pathogen recognition receptors activated by conserved structural motifs known as pathogen associated molecular patterns which are expressed by bacteria, virus and fungi [22]. Upon activation by exogenous (pathogen derived) or endogenous (tissue derived) ligands, all TLRs are able to activate the NF-κB pathway which leads to the synthesis of proinflammatory cytokines and chemokines [7]. Proinflammatory stimulation of endothelial cells have been to shown to release HMGB1 [23], a ligand to TLR2 and 4; with its release resulting in an increase in expression of cytokines, adhesion molecules and the attraction of inflammatory cells to the site of tissue damage [23], [24]. Therefore, the expression of TLR2 and 4 on the endothelium suggests that secretion of HMGB1 in response to fluctuating glucose concentrations may play a pivotal role in contributing to the inflammatory responses via the NF-ĸB cascade.

To date, the precise mechanisms in which glucose fluctuations mediate inflammation in endothelial dysfunction is unknown. Thus, we hypothesized that the activation of TLR2 and 4 when exposed to glucose fluctuations may provide a mechanistic link between inflammation and endothelial dysfunction in microvascular complications.

## Material and Methods

### Ethics statement

All animal experiments were performed with the approval of the animal ethics committee of the University of Sydney. Experiments were conducted by following established guidelines for animal care and were approved by the animal ethics committee of the University of Sydney.

### In vivo experiments

Wildtype Balb/c mice were obtained from the Animal Resource Centre (Perth, Australia). TLR2^-/-^ mice and TLR4^-/-^ mice on a Balb/c background were provided by the Animal Service of Australian National University and Professor D Hume from the University of Queensland with permission from Professor S Akira (Osaka University, Osaka, Japan). The mice were housed in a specific pathogen-free facility in the University of Sydney. Male mice aged 7–8 weeks were used in all experiments. The mice were housed in individual microisolator cages with free access to sterile acidified water and irradiated food in a specific pathogen–free facility at the University of Sydney. Diabetes was induced in the mice by intraperitoneal injections of streptozotocin (STZ) at a dose of 55 mg/kg in sodium citrate buffer (pH 4.5) for 5 consecutive days at 7 to 8 weeks of age. In all murine models utilized in this *in vivo* study, only mice with blood glucose levels >16 mmol/L were considered diabetic. Diabetic mice received insulin (Lantus, Germany) treatment to prevent ketosis. Animals were culled at 24 weeks post induction of diabetes under isoflurane anaesthesia and cardiac puncture.

### Cell Culture

Human dermal microvascular cells (HMEC-1s) were kindly provided by Prof. Gregory Dusting, from the University of Melbourne, Australia. Dermal microvascular endothelial cells initially obtained from human foreskin and immortalised with a PBR-322-based plasmid containing the coding region for the simian virus 40A gene product, large T-antigen [25], were grown in MCDB-131 medium supplemented with 10% fetal calf serum (FCS; Biochrom, Berlin, Germany), 2 mM glutamine, 100 U/ml penicillin, 100 µg/ml streptomycin, 0.04 mg/ml hydrocortisone, and 10 ng/ml EGF. Cell culture media was changed every 48 hours. These cells were grown at 37°C in a humidified 5% CO_2_ incubator and were subcultured at 50–80% confluence using 0.05% trypsin, 0.02% EDTA (Gibco, NY, USA).

### Experimental Protocol

HMEC-1 cells were cultured for 72 hours under four different glucose conditions: 1) control (5 mM glucose); 2) high (30 mM glucose); and 3) fluctuating glucose, where cells were exposed for 2 hours to medium containing 30 mM glucose followed by 3 hours exposure to 5 mM glucose medium, with this cycle repeated three times a day. Cells were then incubated for 12 hours overnight in 5 mM glucose. This was repeated over the 72 hours experimental period. In this fluctuating protocol, the cells were exposed to a total of 270 mM glucose over a 24 hours period compared with 120 mM under control conditions. 4) Moderately elevated glucose involved constant exposure to 11.2 mM glucose. This condition gave the same total glucose load over 24 hours as the fluctuating condition (270 mM glucose) but delivered in a constant manner. All media were changed simultaneously for all conditions, and media were pre-equilibrated and held at 37°C in the tissue culture incubator for the duration of the experiment. Experiments were performed at the conclusion of the 72 hour experimental protocol.

In order to assess for the effect of HMGB1 on NF-ĸB activation, HMEC-1 cells were stimulated with 500 ng/ml of recombinant HMGB1 (Protein One).

### Western blot

Cells collected were 95% confluent and the cell pellet was resuspended in cell lysis buffer containing 50 mM Tris-HCl, 150 mM NaCl, 5 mM EDTA (pH 7.4), 0.5% TritonX-100 and protease inhibitors (Roche Diagnostics, Manheim, Germany). Cell lysate was spun at 13 000 rpm at 4°C for 5 minutes and stored at −20°C. Protein quantification (Bio-Rad, CA, USA) was carried out to determine the protein concentration of the cell lysate. 50 µg total cell protein was mixed with 6X Laemmli sample buffer containing β-mercaptoethanol and heated at 95°C for 10 minutes. Samples were then analyzed by sodium dodecyl sulphate polyacrylamide gel electrophoresis (SDS-PAGE) and electroblotted to Hybond Nitrocellulose membranes (Amersham Pharmacia Biotech, Bucks, UK). Membranes were blocked in Tris-buffered saline containing 0.2% Tween-20 (TBST) in 5% skim milk for 2 hours and then incubated overnight at 4°C with the following antibodies – TLR2 1∶500 (Imgenex, San Diego, CA), TLR4 1∶125 (Invitrogen, CA, USA), HMGB1 1∶1000 (Abcam, MA, USA), NF-ĸB p65 1∶200 (SantaCruz, CA, USA), ICAM-1 1∶1000 (Cell Signalling, MA, USA), VCAM-1 1∶500 (Abcam, MA, USA). Membranes were washed with TBST and incubated with horseradish peroxidise conjugated secondary antibody. Proteins were visualised using the enhanced chemiluminescence (ECL) detection system (Amersham Pharmacia Biotech, Bucks, UK). All membranes were reprobed with β-actin 1∶300 (Santa Cruz, CA, USA) and results were corrected for actin as a loading control and analysed using ImageJ software (Java based software program, NIH).

### Nuclear extraction and EMSA

Nuclear extracts were prepared using NucBuster Protein Extraction Kit (Novagen, Darmstadt, Germany) as per manufacturer's instructions. The DIG Gel Shift Kit (Roche Applied Science, Indianapolis, US) was used in the EMSA. 25 µg of nuclear extract were incubated with 1 ug poly [d (I-C)] as the non specific competitor, 1 µg poly L-lysine in a binding buffer (100 mM Hepes, pH 7.6, 5 mM EDTA, 50 mM (NH_4_)SO_4_, 5 mM DTT, Tween 20, 1% w/v, 150 mM KCl) and dig-labelled NF-ĸB (5′-AGT TGA GGG GAC TTT CCC AGG C-3′) consensus oligonucleotide (Promega, WI, USA) for 30 minutes at room temperature. The reaction mixture was electrophoresed through 6% polyacrylamide gels, transferred onto nylon positively charged membrane (Roche Applied Science, Indianapolis, USA) and then crosslinked using an UV-transilluminator for 3 minutes. The membrane was subjected to immunological detection using anti-Digoxigenin-AP conjugate and chemiluminescence. Results were analyzed using Image J software and shift bands were quantified.

### Real-time PCR

RNA was extracted using an RNeasy mini kit (Qiagen, Valencia, CA) according to manufacturer's instructions. RNA was then DNAsed and RT-PCR was performed with SuperScriptIII One-Step RT-PCR System and Platinum Taq DNA polymerase (Invitrogen). cDNA was generated by reverse transcribing 1 µg of total RNA in a reaction volume of 20 µl using VILO cDNA synthesis kits (Invitrogen). One microliter (50 ng) of cDNA was used as a template in a 20 µl PCR reaction. Quantitative real-time PCR was performed using an ABI Prism 7900 HT Sequence Detection System (Applied Biosystems, Foster City, CA) with Taqman Gene Expression Master Mix (Applied Biosystems) and gene-specific expression assays containing two unlabelled PCR primers. Reactions were performed in at least triplicate and were analyzed by relative quantitation using RQ Manager software, version 1.2 (Applied Biosystems). The following primers were used for mRNA detection: MCP-1: Forward 5′- CCAAAGAAGCTGTGATCTTCAA Reverse 5′- TGGAATCCTGAACCCACTTC, IL-8: Forward 5′- AGCCTTCCTGATTTCTGCAGGCT Reverse 5′- AATTTCTGTGTTGGCGCAGTGTGG, ICAM-1: Forward 5′- TGTGGTAGCAGCCGAGTCATAAT Reverse- CGTGGCTTGTGTGTTCGGTTTCAT, Actin: Forward 5′- GCTCGTCGTCGACAACGGCCTC Reverse 5′- CAAACATGATCTGGGTCATCTTCTC. All data are presented as fold-change compared with control after normalization to β-actin.

### TLR2 signalling blockade with a neutralizing antibody

Anti-TLR2-IgA (Invivogen, CA, USA) is a chimeric monoclonal antibody specific for human TLR2. It was generated by combining the constant domains of the human IgA molecule with murine variable regions. Anti-TLR2-IgA has been selected for its ability to efficiently neutralize the biological activity of TLR2 by blocking TLR2 agonists-induced cellular activation. In this study, anti-TLR2-IgA was dissolved in sterile water. HMEC-1 cells were seeded into 6 well plates. After 24 hours, cells were incubated with 0.5 µg/ml of anti-TLR2-IgA and 500 ng/ml recombinant HMGB1 for 2 hours. The negative control utilized in this experiment was an appropriate isotype control product (Human IgA2 isotype control, Invivogen). Thereafter, nuclear extract and protein was harvested and NF-ĸB-DNA binding activity and NF-ĸB p65 expression was determined.

### TLR4 signalling inhibition with an intracellular signalling inhibitor

TAK-242 (resatorvid, ethyl (6R)-6-[N-(2-chloro-4-fluorophenyl)sulfamoyl] cyclohex-1-ene-1-carboxylate] was synthesized at Takeda Pharmaceutical Company Limited (Osaka, Japan). TAK-242 suppresses ligand-induced NF-ĸB activation with a 50% inhibitory concentration (IC_50_) of 110 nM. In this study, TAK-242 was dissolved in dimethyl sulfoxide (DMSO). HMEC-1 cells were seeded into 6 well plates. After 24 hours, cells were incubated with TAK-242 at 1 µM or vehicle control (DMSO) for 2 hours. To upregulate the expression of NF-ĸB, cells were exposed to 500 ng/ml recombinant HMGB1 (ProteinOne) for 2 hours. Thereafter, nuclear extract and protein was harvested and NF-ĸB-DNA binding activity and NF-ĸB p65 expression was determined.

### MCP-1 and IL-8 ELISA

HMEC-1 cells were seeded in 6-well plates and were exposed to the experimental conditions as defined above in triplicate. After treatment, the supernatant was removed and centrifuged at 13 000 rpm for 5 minutes. Protein concentrations of secreted MCP-1 and IL-8 were determined using commercially available ELISA kits (R&D systems and Invitrogen) as per manufacturer's instructions. The optical density (OD) at 450 nm was then read using a microplate reader. Cell lysate protein concentration from corresponding wells was determined by protein assay (Biorad). MCP-1 and IL-8 levels were corrected for protein content per well.

### Immunohistochemistry

Formalin-fixed paraffin-embedded section of 4 µM were deparaffinised and boiled for 10 minutes in 10 mM sodium citrate buffer (pH 6.0). Immunohistochemistry was performed using goat anti-mouse ICAM-1 (R&D Systems, MN, USA) antibody. Concentration-matched goat IgG was used as isotype-negative control. Sections were exposed to 8% H_2_0_2_ for 5 minutes to quench endogenous peroxidises. The sections were blocked with serum-free protein block (Dako) for 10 minutes and incubated with anti-ICAM-1 primary antibody for 1 hour at room temperatur. Slides were then incubated with biotinylated secondary antibody anti-goat (Dako) for 1 hour at room temperature. Following, secondary antibody incubation, slides were incubated with VECTASTAIN ABC kit (Vector Laboratories) followed by DAB substrate-chromogen solution. Thereafter, the slides were counterstained with Harris hematoxylin.

The tissue specimens were examined by bright field microscopy using a Leica photomicroscope linked to a DFC 480 digital camera.

The quantitation of ICAM-1 staining was performed by capturing 10 random non-overlapping fields from stained sections. Areas of brown staining reflecting ICAM-1 were highlighted using a selective colour tool for their colour ranges and the proportional area of their tissue with their respective ranges of colour was then quantified. Glomerular expression of ICAM-1 was assessed as percent area stained within the glomerular tuft. Calculation of the proportional area stained brown was determined using the automated cellular imaging system Image J.

### Statistical Analyses

Normalized results are expressed as a percentage of the mean + standard error of control values or as stated. Experiments were performed at least in triplicate or as detailed in text. Statistical comparisons between groups were made by using unpaired Student T-tests or Mann-Whitney. Analyses were performed using the software package, Statview version 5.0 (Abacus Concepts Inc., Berkley, CA, USA). *P* values <0.05 were considered significant.

## Results

### Maximal expression of TLR4 with fluctuating glucose

**Figure 1:**
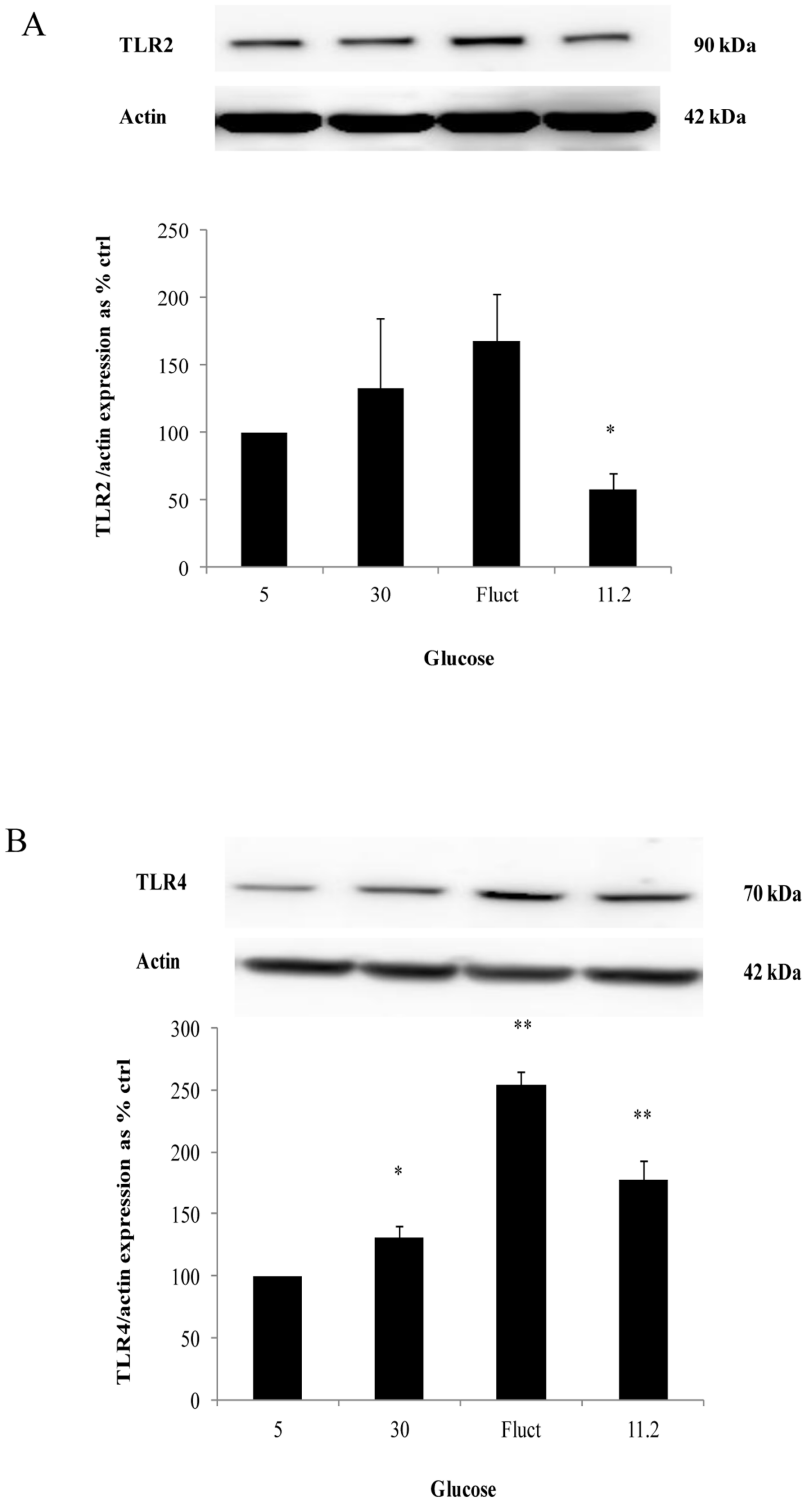
TLR2 and 4 expression in cells exposed to the defined experimental conditions for 72 hours. (A) Reduction in TLR2 expression with 11.2 mM glucose and (B) maximal TLR4 expression with fluctuating glucose conditions. Normalized results are expressed as mean ± SEM, n = 4. *P<0.05 versus HMEC-1 cells cultured with 5 mM glucose. **P<0.01 versus HMEC-1 cells cultured with 5 mM glucose.

HMEC-1 cells were exposed to 5 mM, 30 mM, fluctuating and 11.2 mM glucose for 72 hours and assessed for TLR2 and TLR4 expression. Although there was a maximal increase in TLR2 expression in the fluctuating glucose limb, this did not reach statistical significance. TLR2 expression was reduced to 58.1±11.3% (*P*<0.05) of control values with 11.2 mM glucose (Figure 1A).

An increase in TLR4 expression was shown in all limbs exposed to high glucose with the maximal increase noted in the fluctuating glucose limb, being 253.7±10.9% (*P*<0.01) of control levels. 30 mM and 11.2 mM glucose increased TLR4 expression to 130.9±9.82% (*P*<0.05) and 177.0±16.4% (*P*<0.01) of control values respectively (Figure 1B).

### Maximal increase in NF-ĸB activation with fluctuating glucose

**Figure 2:**
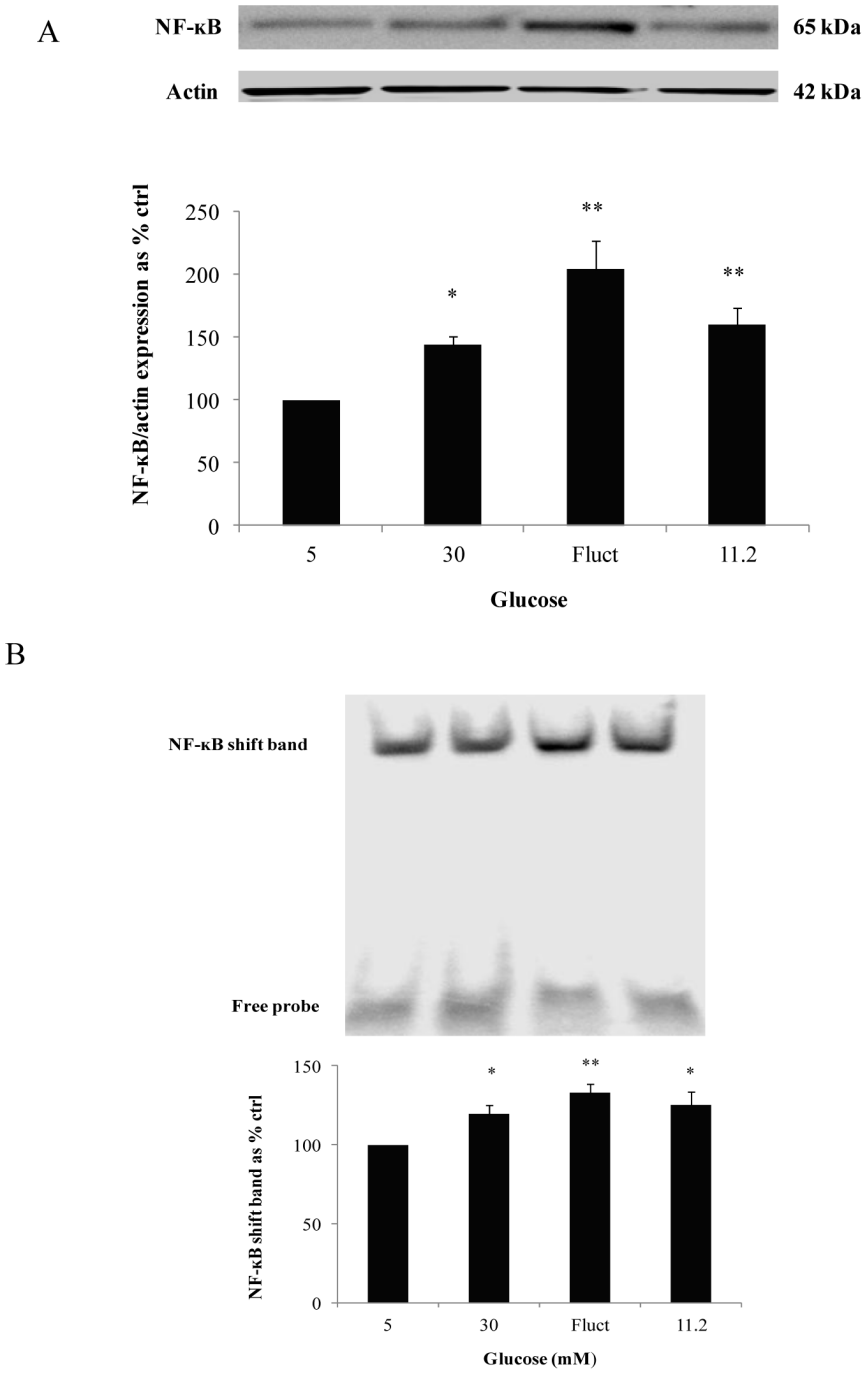
Expression of NF-ĸB p65 and NF-ĸB-DNA binding from cells exposed to experimental conditions for 72 hours. (A) Maximal increase in nuclear NF-ĸB p65 subunit expression was observed in fluctuating glucose limb and, (B) concomitant maximal increase in NF-ĸB-DNA binding was detected in cells with exposure to fluctuating glucose conditions for 72 hours. Normalized results are expressed as mean ± SEM, n = 3. *P<0.05 versus HMEC-1 cells cultured with 5 mM glucose. **P<0.01 versus HMEC-1 cells cultured with 5 mM glucose.

NF-ĸB, a downstream target of TLRs in the inflammatory pathway was subsequently determined. Consistent with the maximal increase observed in TLR2 and TLR4 expression we observed maximal increase in nuclear NF-ĸB p65 subunit expression in the fluctuating limb, the values being increased to 204.9±21.6% (*P*<0.01) of control values. Furthermore, exposure to 30 mM glucose and 11.2 mM glucose also increased nuclear NF-ĸB p65 subunit expression to 144.3±6.93% (*P*<0.05) and 159.7±13.8% (*P*<0.01) of control values respectively (Figure 2A).

Similar to NF-ĸB p65 subunit expression, a maximal increase of 133.0±5.56% (*P*<0.01) in nuclear NF-ĸB-DNA binding of control levels was observed in the fluctuating limb. NF-ĸB-DNA binding was also increased in the 30 mM and 11.2 mM glucose limbs, being 119.4±5.97% (*P*<0.05) and 124.8±8.94% (*P*<0.05) of control values respectively (Figure 2B).

### Changes in proinflammatory cytokines and cell adhesion molecules

MCP-1 transcription did not parallel NF-ĸB expression. On the contrary, a significant reduction in MCP-1 transcription was observed in the fluctuating glucose limb, being 0.736±0.06-fold (*P*<0.01) of control values.

**Figure 3:**
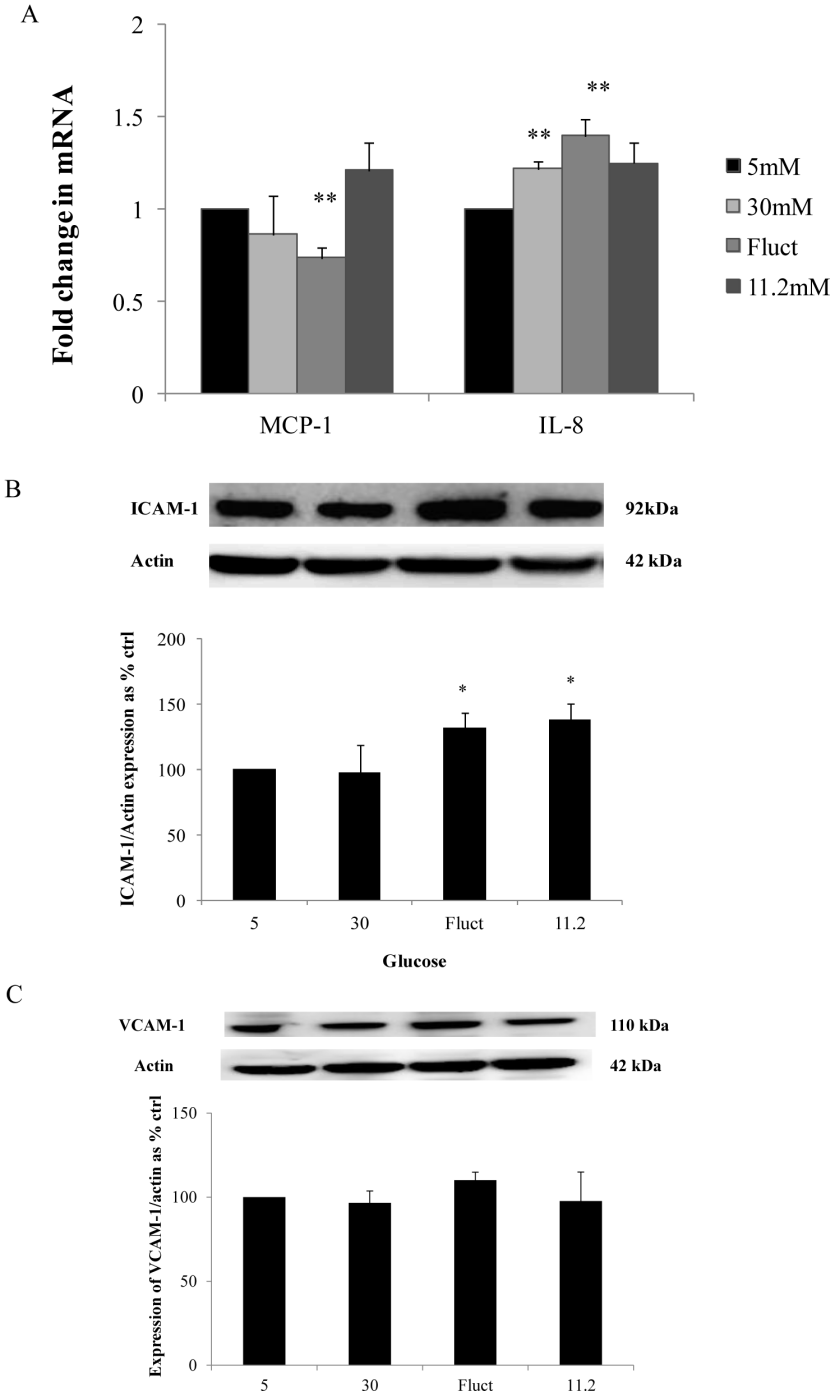
Expression of cytokines and cell adhesion molecules with exposure to defined conditions for 72 hours. (A) Reduction in MCP-1 transcription was detected in the fluctuating glucose limb whereas an increase in IL-8 transcription was detected in the 30 mM glucose and fluctuating glucose limbs with maximal increase in cells exposed to fluctuating glucose concentrations for 72 hours. (B) ICAM-1 protein expression increased in both the fluctuating and 11.2 mM glucose limbs however; (C) VCAM-1 expression was not induced by any experimental condition. Normalized results are expressed as mean ± SEM, n = 4. *P<0.05 versus HMEC-1 cells cultured with 5 mM glucose. **P<0.01 versus HMEC-1 cells cultured with 5 mM glucose.

Conversely, IL-8 transcription was increased both in the 30 mM glucose and fluctuating glucose limbs. Exposure to 30 mM glucose induced IL-8 transcription to 1.22±0.04-fold (*P*<0.01) of control levels and in keeping with the proinflammatory environment generated by the effects of fluctuating glucose concentrations in HMEC-1 cells, we detected a maximal increase in IL-8 transcription in the fluctuating glucose limb, being 1.39±0.09-fold (*P*<0.01) of control levels (Figure 3A).

Cell adhesion molecules play an integral role in transducing inflammation in endothelial cells. There was a significant increase in ICAM-1 expression in both the fluctuating glucose and 11.2 mM glucose limbs being 132.0±11.9% (*P*<0.05) and 138.3±12.0% (*P*<0.05) of control values respectively (Figure 3B). However, there was no significant increase in VCAM-1 expression in all glucose limbs (Figure 3C).

### Increase in secreted HMGB1 with high glucose concentrations and increase in HMGB1 expression in the fluctuating glucose limb

**Figure 4:**
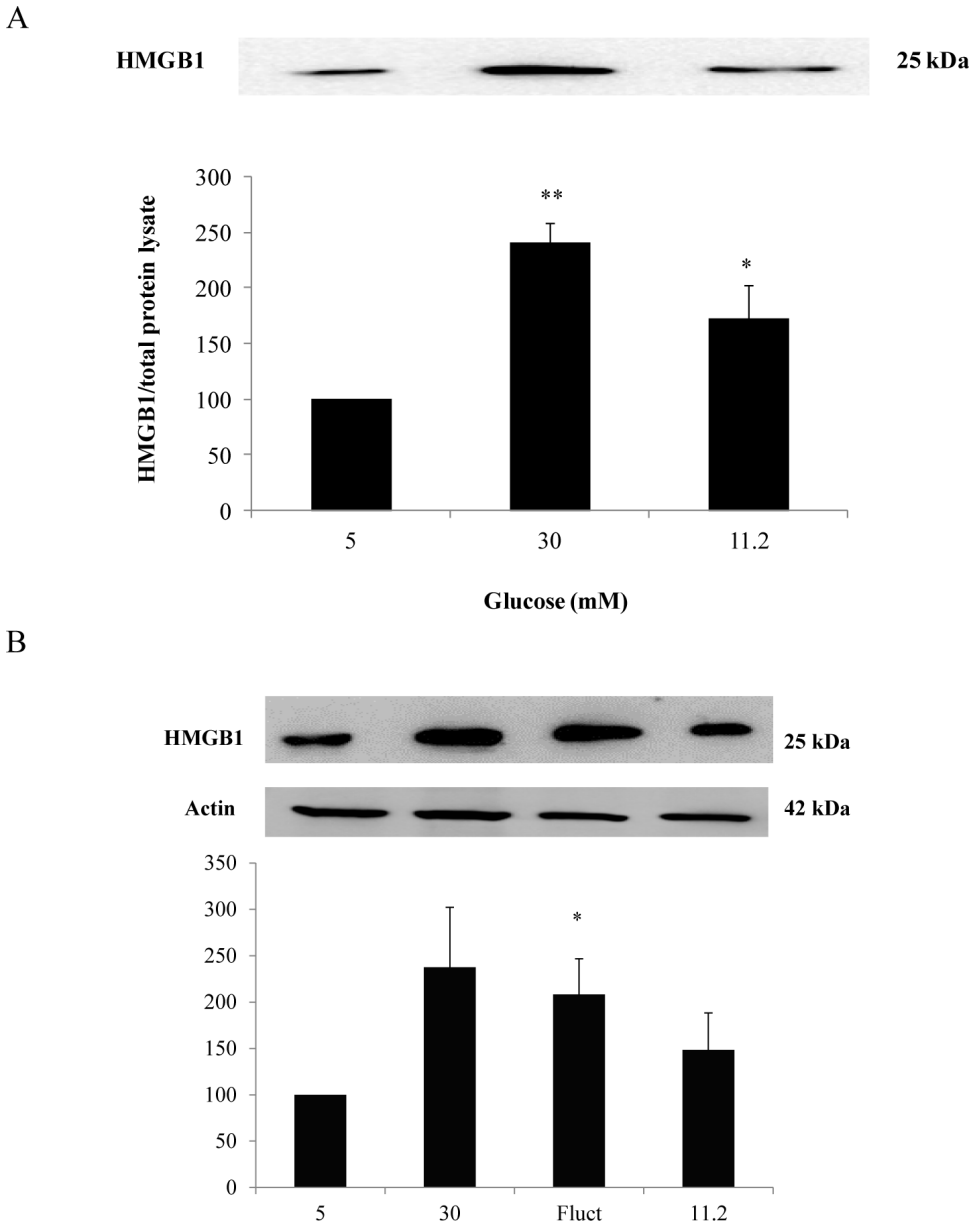
Expression of secreted HMGB1 with exposure to high and moderate glucose concentrations for 72 hours. (A)An increase in secreted levels of HMGB1 was detected in the supernatant of cells exposed to high glucose levels of 30 mM and 11.2 mM glucose for 72 hours. Western blot analysis was performed on equal volumes of supernatant and normalized to total protein in corresponding cell lysates. (B) HMGB1 protein expression was increased in the fluctuating glucose limb. No significant increase in HMGB1 expression was observed with exposure to high and moderate levels of glucose. Normalized results are expressed as mean ± SEM, n = 3. *P<0.05 versus HMEC-1 cells cultured with 5 mM glucose. **P<0.01 versus HMEC-1 cells cultured with 5 mM glucose.

HMGB1 is a proinflammatory cytokine that plays a critical role in endothelial dysfunction [26], [27]. Here, we determined the presence of HMGB1 in the supernatants of HMEC-1 cells cultured with 30 mM and 11.2 mM glucose for 72 hours without a change in media. With 30 mM and 11.2 mM glucose, there was an upregulation of HMGB1 protein secretion expression to 240.9±18.1% (*P*<0.01) and 172.5±23.0% (*P*<0.05) of control values respectively (Figure 4a). It is not feasible to determine the release of HMGB1 into the supernatant with fluctuating glucose due to the change of media every 2–3 hours. However, we also assessed for HMGB1 protein expression in cell lysate and observed an increase of 208.2±38.6% (*P*<0.05) of control values in the fluctuating glucose limb. Although there was also an increase HMGB1 protein expression in the other glucose limbs this did not reach statistical significance (Figure 4B).

### NF-ĸB is activated with recombinant HMGB1 stimulation

**Figure 5:**
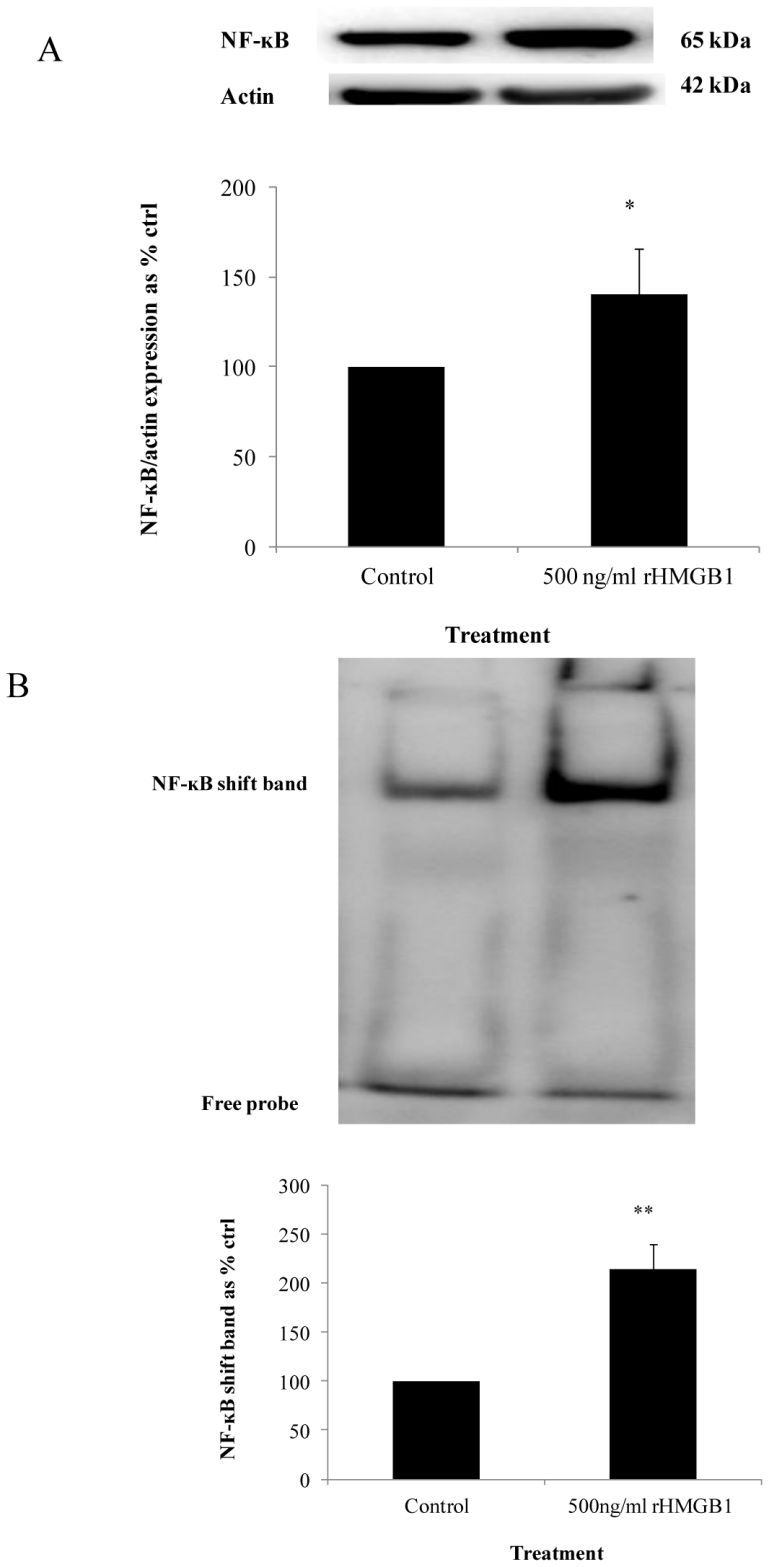
The effect of recombinant HMGB1 on NF-ĸB p65 subunit expression and NF-ĸB-DNA binding. (A) Exposure to recombinant HMGB1 (500 ng/ml) in control media for 2 hours induced nuclear NF-ĸB p65 subunit expression and, (B) NF-ĸB-DNA binding was also induced with exposure to recombinant HMGB1 (500 ng/ml) in HMEC-1 cells. Normalized results are expressed as mean ± SEM, n = 5. *P<0.05 versus HMEC-1 cells cultured in the absence of recombinant HMGB1. **P<0.01 versus HMEC-1 cells cultured in the absence of recombinant HMGB1.

HMGB1 is known to stimulate NF-ĸB activation in inflammation. We exposed HMEC-1 cells to 500 ng/ml of recombinant HMGB1 for 2 hours to deduce the role of HMGB1 in endothelial cells. There was a significant increase in nuclear NF-ĸB p65 subunit expression to 140.4±25.2% (*P*<0.05) of control values (Figure 5A) and NF-ĸB-DNA binding to 214.4±25.9% (*P*<0.01) of control values in the presence of recombinant HMGB1 (Figure 5B).

### NF-ĸB activation induced MCP-1, IL-8 and ICAM-1 expression

**Figure 6:**
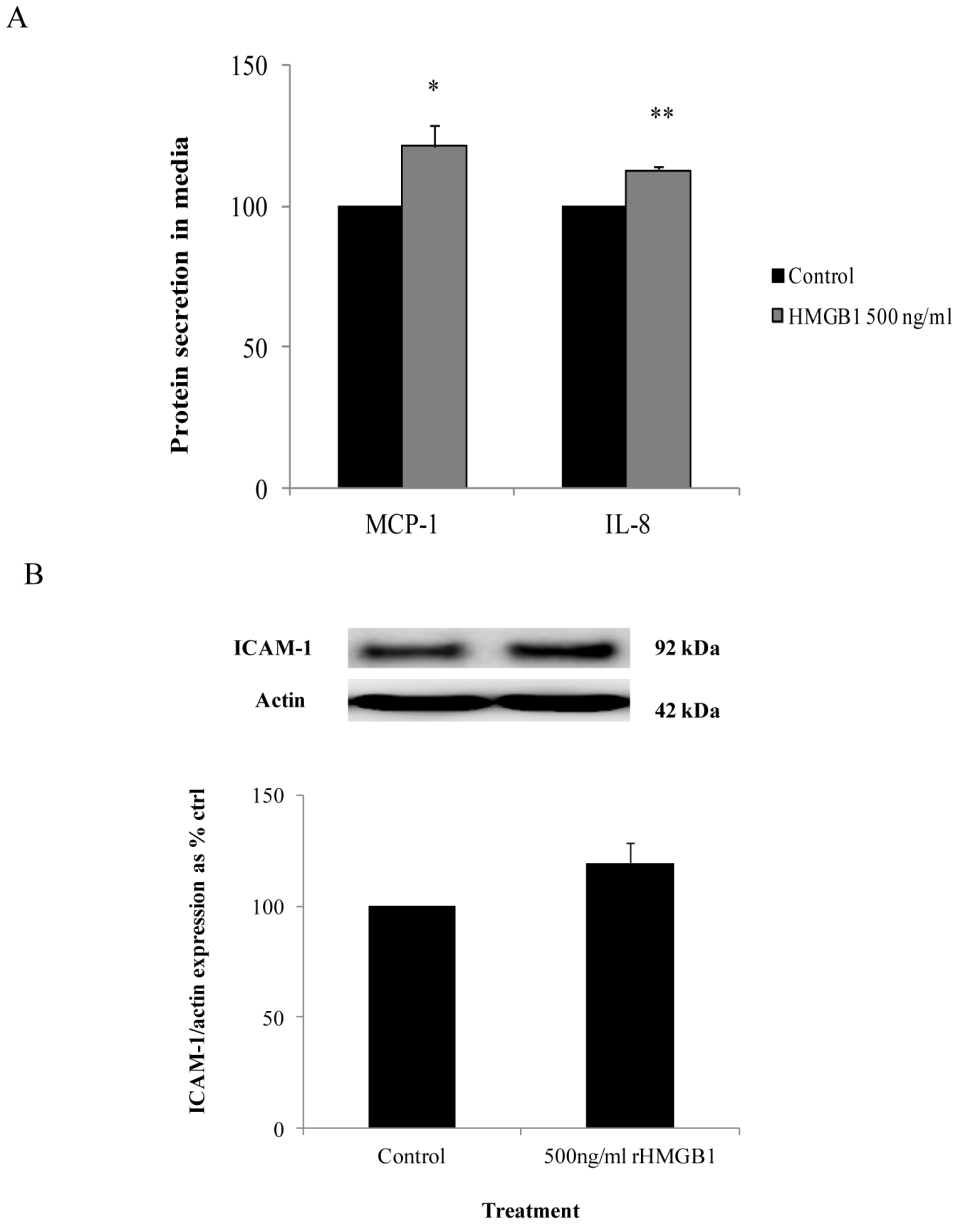
The effect of recombinant HMGB1 on stimulating inflammatory cytokines and cell adhesion molecules. (A) Stimulating HMEC-1 cells with recombinant HMGB1 in control media for 2 hours induced the secretion of MCP-1 and IL-8 into the media (B) Exposure to recombinant HMGB1 also induced a moderate increase in ICAM-1. Normalized results are expressed as mean ± SEM, n = 3. *P<0.05 versus HMEC-1 cells cultured in control media. **P<0.01 versus HMEC-1 cells cultured in control media.

Recombinant HMGB1 induced MCP-1 protein secretion to 121.2±7.59% (*P*<0.05) of control values and IL-8 secretion to 112.7±1.43% (*P*<0.01) of control values respectively (Figure 6A). Recombinant HMGB1 also induced ICAM-1 expression to 119.2±9.22% (*P* = 0.05) of control values (Figure 6B).

### The effect of inhibiting TLR2, TLR4 or both signalling pathways on NF-ĸB activation

**Figure 7:**
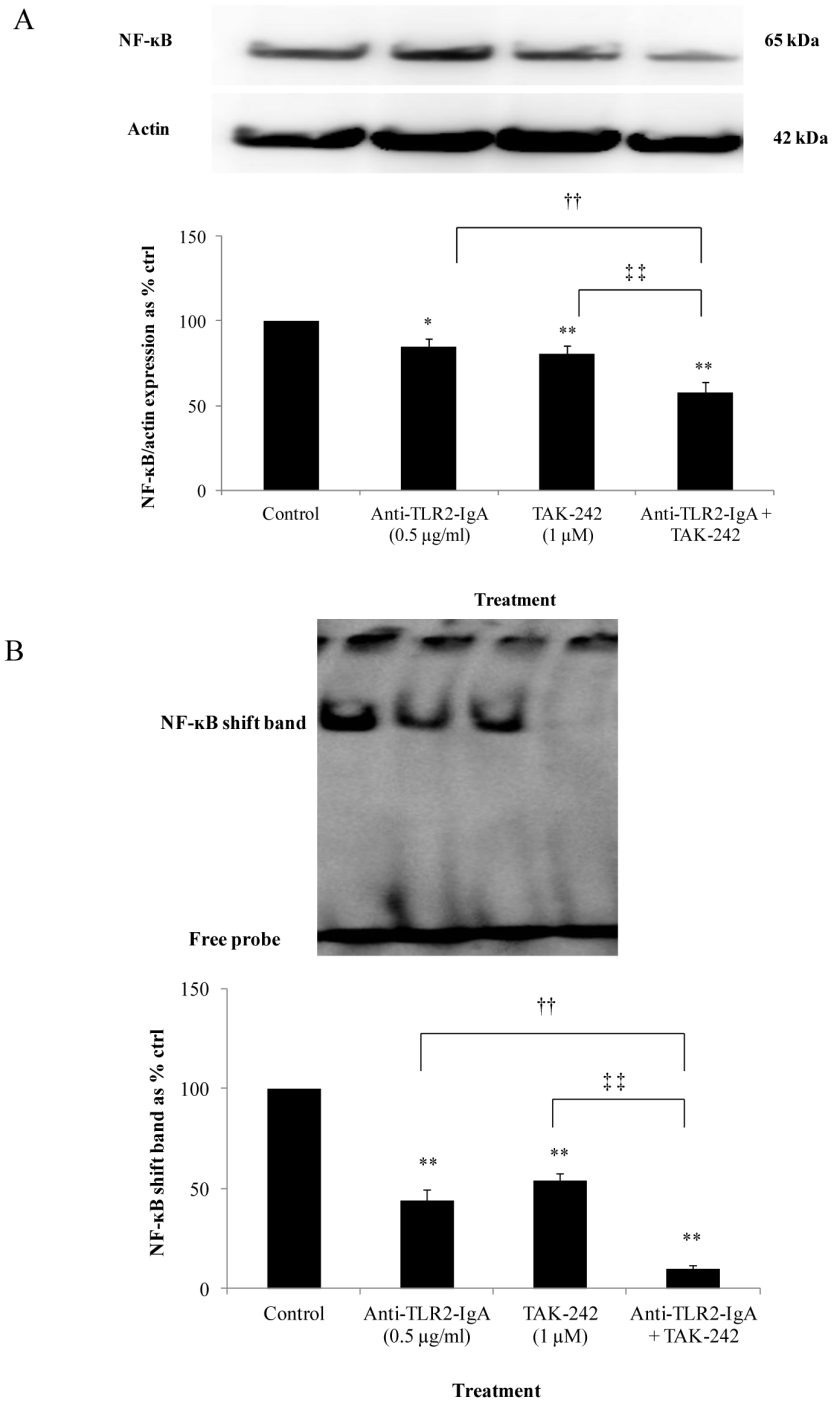
Effect of separate and additive inhibition of TLR2 and TLR4 signalling pathways on NF-ĸB activation. (A) Reduction in NF-ĸB p65 subunit expression when TLR2 and 4 inhibitors were used separately and further abrogation of NF-ĸB p65 subunit expression when the inhibitors were used additively in control media (B) An attenuation in NF-ĸB-DNA binding was observed when anti-TLR2-IgA and TAK-242 were used separately and further downregulation in NF-ĸB-DNA binding when both inhibitors were used additively in control media. Normalized results are expressed as mean ± SEM, n = 4. *P<0.05 versus HMEC-1 cells treated with DMSO + control IgA (control). **P<0.01 versus HMEC-1 cells treated with DMSO + control IgA (control). ^††^P<0.01 versus HMEC-1 cells treated with Anti-TLR2-IgA. ^‡‡^P<0.01 versus HMEC-1 cells treated with TAK-242.

The effect of TLR2 and TLR4 signalling interruption as well as the dual inhibition of both pathways was determined on NF-ĸB activation by the use of a TLR2 neutralizing antibody and a TLR4 signalling inhibitor TAK-242. When TLR2 neutralizing antibody and TAK-242 were used separately and then stimulated with recombinant HMGB1, NF-ĸB p65 subunit expression was attenuated to 84.6±4.92% (*P*<0.05) and 80.8±4.51% (*P*<0.01) of control values respectively. However, when used together, the additive effect of anti-TLR2-IgA and TAK-242 further downregulated NF-ĸB p65 subunit expression to 57.7±6.12% (*P*<0.01) of control values (Figure 7A).

Similarly, when TLR2 neutralizing antibody and TAK-242 were used separately, NF-ĸB-DNA binding was reduced to 43.8±5.62% (*P*<0.01) and 53.8±3.57% (*P*<0.01) of control values respectively. When used in conjunction, the dual inhibition resulted in a further attenuation of NF-ĸB-DNA binding to 9.54±2.23% (*P*<0.01) of control values. The dual inhibition significantly downregulated NF-ĸB-DNA binding compared to the individual use of TLR2 neutralizing antibody (*P*<0.01) or TAK-242 (*P*<0.01) (Figure 7B).

**Figure 8:**
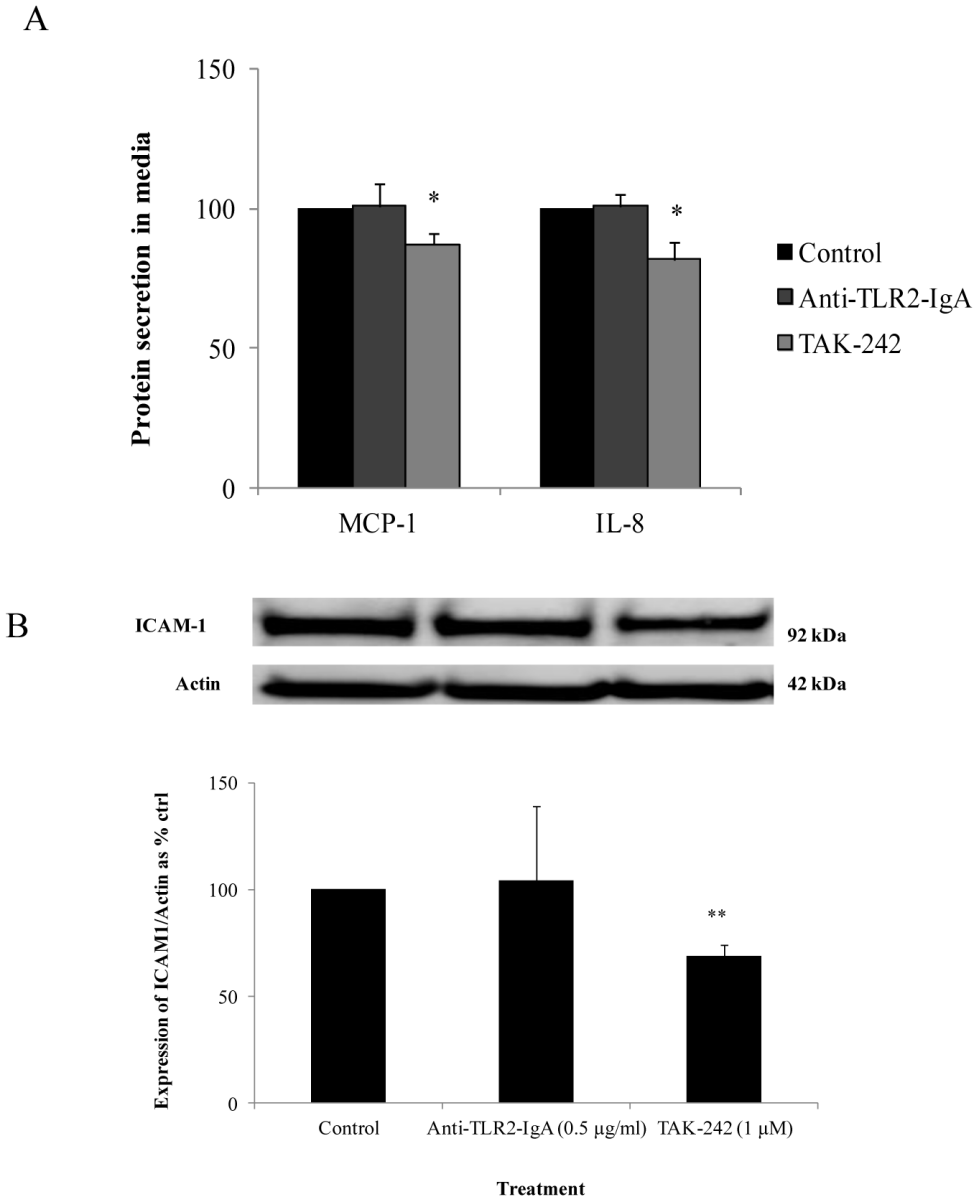
Effect of anti-TLR2-IgA or TAK-242 on inflammatory cytokines and cell adhesion molecules. (A) With exposure to TLR2 neutralizing antibody in control media, there was no reduction in MCP-1 and IL-8 expression however with exposure to TAK-242 in control media, there was a suppression in MCP-1 and IL-8 expression (B) With exposure to TLR2 neutralizing antibody in control media, there was no reduction in ICAM-1 expression but TAK-242 suppressed ICAM-1 expression. Normalized results are expressed as mean ± SEM, n = 3. **P<0.01 versus control.

The release of pro-inflammatory cytokines was measured when HMEC-1 cells were exposed to TLR2 neutralizing antibody and TAK-242. Following treatments with the respective inhibitors, HMEC-1 cells were exposed to recombinant HMGB1. There was no significant change in MCP-1 and IL-8 protein secretion with TLR2 neutralizing antibody but with TAK-242, there was a reduction in MCP-1 and IL-8 secretion to 87.2±3.65% (*P*<0.05) and 81.8±6.37% (*P*<0.05) of control values respectively (Figure 8A). Since there was no reduction with TLR2 inhibition, a combination of TLR2 and 4 inhibition was not pursued.

With treatment with TLR2 neutralizing antibody, there was no change in ICAM-1 expression however with TAK-242, there was a reduction in ICAM-1 expression to 69.1±4.8% (*P*<0.01) of control values respectively (Figure 8B).

### ICAM-1 expression in wildtype, TLR2^-/-^ and TLR4^-/-^ mice

**Figure 9:**
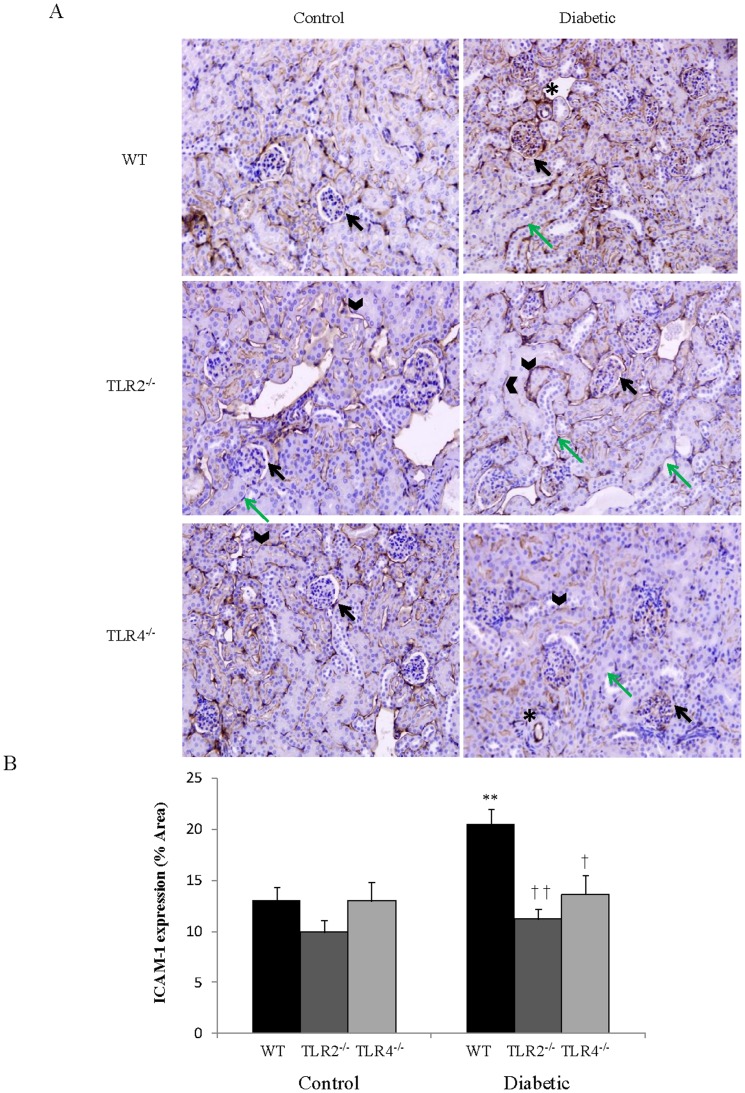
ICAM-1 expression in wildtype, TLR2^-/-^ and TLR4^-/-^ murine models. (A) In the wildtype, TLR2^-/-^ and TLR4^-/-^ murine models, ICAM-1 was expressed in the glomeruli (black arrows), peritubular capillaries (asterisks), epithelial cells (green arrows) and tubular brush border (arrowheads) (B) There was a significant upregulation of ICAM-1 in the glomeruli of wildtype diabetic mice compared to wildtype mice without diabetes. This upregulation was attenuated in TLR2^-/-^ and TLR4^-/-^ diabetic mice compared to wildtype diabetic mice. *P<0.01 versus wildtype mice without diabetes. ^†^P<0.05 versus wildtype mice induced with diabetes. ^††^P<0.01 versus wildtype mice induced with diabetes.

To determine the role of TLR2 and 4 in the pathogenesis of microangiopathy, we assessed for ICAM-1 expression in TLR2^-/-^ and TLR4^-/-^ murine models after the induction of diabetes. Immunohistochemistry revealed that ICAM-1 was expressed in basal levels in glomeruli, peritubular capillaries and the tubular brush border in non-diabetic wildtype mice (Figure 9A). There was no significant difference in ICAM-1 expression between wildtype, TLR2^-/-^ and TLR4^-/-^ mice without diabetes (control groups). However, with the induction of diabetes, there was a marked upregulation in ICAM-1 expression in the glomeruli. Moreover, in TLR2^-/-^ and TLR4^-/-^ mice induced with diabetes, there was an amelioration of glomeruli ICAM-1 expression versus wildtype diabetic mice (Figure 9B).

## Discussion

In this study, we have clearly shown that HMEC-1 cells exposed to fluctuating glucose concentrations demonstrated an enhanced inflammatory response with an increase in the expression of TLR4. The inflammatory phenotype of HMEC-1 cells in the presence of fluctuating glucose was characterized by augmented NF-ĸB activation and the concomitant synthesis of cytokines and cell adhesion molecules. We have also shown that high glucose concentrations upregulated HMGB1, a ligand to TLR2 and 4 known to transduce inflammation through NF-ĸB activation in HMEC-1 cells [24], [28]. With exposure to recombinant HMGB1, we demonstrated NF-ĸB activation and an increase in MCP-1, IL-8 and ICAM-1 expression in HMEC-1 cells. Furthermore, we have uniquely demonstrated a downregulation in HMGB1-mediated NF-ĸB activation in HMEC-1 cells when TLR2 and 4 signalling pathways were inhibited and this was additive when dual inhibition took place. With the inhibition of TLR4 but not TLR2 signalling pathway, we observed attenuation in downstream inflammatory markers including MCP-1, IL-8 and ICAM-1 expression. Our findings demonstrate that although both TLR2 and TLR4 are involved in the activation of transcription factor NF-ĸB when exposed to high and moderate glucose, it is likely that TLR4 may be the more pathogenic receptor in sustaining the downstream pro-inflammatory cascasde in diabetic microangiopathy. Additionally, postprandial glucose fluctuations may synergistically be amplifying inflammatory responses in the human microvasculature through the activation of TLR4.

There is a paucity of data examining the role of TLR2 and 4 in glucose and HMGB1 induced endothelial dysfunction. In this study, we present novel findings that fluctuating glucose conditions induced TLR4 expression greater than 30 mM or 11.2 mM glucose concentrations in human microvascular endothelial cells. This maximal TLR4 expression in the fluctuating glucose limb also correlated to maximal increase in NF-ĸB activation and downstream expression of chemokines and cell adhesion molecules implicating the involvement of TLR4 in vascular complications of diabetes. Although our *in vitro* studies do not demonstrate an increase in TLR2 expression with exposure to the different glucose levels, we demonstrated a reduction in ICAM-1, a key regulator of endothelial inflammation [29] in TLR2 knockout diabetic mice. *Li et al.*, had also demonstrated the involvement of both TLR2 and 4 in inducing inflammation via NF-ĸB activation in diabetic coronary artery endothelial cells [30].

Downstream to NF-ĸB activation, we showed increased expression of cytokines and cellular adhesion molecules including IL-8 and ICAM-1 in the fluctuating limb. Importantly we showed dissociation in MCP-1 transcription and NF-ĸB activation with fluctuating glucose. This is in keeping with prior studies done in our lab with high glucose [31], [32], thus, we suggest that MCP-1 may be at least in part governed by non NF-ĸB pathways. With respect to VCAM-1, there was no increase in protein expression with fluctuating glucose. Consistent with our studies, there is no direct evidence thus far that the blockade of VCAM-1 by antibodies or deletion of the genes ameliorating the progression of diabetic nephropathy in animal models. Moreover, insulin was shown to be strongly associated with soluble ICAM-1 and other markers of inflammation such as C-reactive protein and IL-6 whereas soluble VCAM-1 was not, indicating that inflammation and ICAM-1 are an integral part of insulin resistance, further implicating its role in diabetic complications [33].

Supporting TLR2 and 4's involvement in potentiating inflammation, another group has shown that patients with Type 1 diabetes mellitus and microvascular complications exhibited augmented expression of TLR2, 4 and biomarkers of inflammation in their monocytes compared to patients without microvascular complications [20]. Our data provides further evidence that the microvascular endothelium itself may possess a critical role in regulating inflammation in diabetic microangiopathy with postprandial glucose fluctuations contributing a greater part in synergistically increasing the expression of TLR4, cytokines, chemokines and cell adhesion molecules.

We have also shown that HMGB1, a ligand to TLR2 and 4 was secreted by HMEC-1 cells in response to 30 mM and 11.2 mM glucose. Consistent with our results, *Yao et al*., have also shown an increase in HMGB1 secretion when human aortic endothelial cells were exposed to high glucose concentrations [34]. Additionally, we showed an increase in cellular HMGB1 expression in the fluctuating glucose limb indicating the potential involvement of HMGB1 in regulating downstream TLR signalling with fluctuating glucose concentrations. With the further use of recombinant HMGB1, we have illustrated HMGB1 mediated NF-ĸB activation and the concurrent expression of proinflammatory cytokines and cell adhesion molecules including MCP-1, IL-8 and ICAM-1 which are known to be involved in the pathogenesis of inflammation in the endothelium.

Additionally, our data demonstrated that HMGB1 induced NF-ĸB activation in HMEC-1 cells is mediated by both TLR2 and 4. We have demonstrated that the blockade of TLR2 cellular activation with a TLR2 neutralizing antibody (Anti-TLR2-IgA) or the inhibition of TLR4 intracellular signalling with the use of an inhibitor (TAK-242) attenuated HMGB1-mediated NF-ĸB activation in HMEC-1 cells. Our findings are in agreement with *Bae et al*., in which they also demonstrated attenuated NF-ĸB activation in the presence of TLR2 and 4 siRNAs [35]. In addition, we showed that dual inhibition of TLR2 and 4 further ameliorated NF-ĸB activation. However, TLR4 was the more predominant receptor in attenuating the downstream expression of MCP-1, IL-8 and ICAM-1 with no difference observed in the expression of the respective inflammatory markers with a TLR2 neutralizing antibody.

Our data also showed that ICAM-1, a critical mediator of inflammation in diabetic nephropathy [36] was found to be widely expressed in basal levels in the glomeruli, tubular brush border, peritubular capillaries, blood vessels and in addition on some of the renal proximal tubular epithelial cells in wildtype Balb/c mice. There is established evidence that increased ICAM-1 expression is associated with disease progression in diabetic nephropathy [37]–[41] with its genetic deficiency proven to exert renoprotective effects [42], [43]. With the induction of diabetes, we observed a significant upregulation in ICAM-1 expression in the glomeruli of wildtype Balb/c mice, implicating the contribution of hyperglycemia to the pathogenesis of microvascular inflammation in diabetic nephropathy.

Moreover, we have uniquely shown that there is a marked reduction in glomerular ICAM-1 expression in TLR2^-/-^ and TLR4^-/-^ murine models compared to wildtype mice induced with diabetes. Additionally, there was no significant difference in glomerular ICAM-1 in diabetic TLR2^-/-^ and TLR4^-/-^ mice compared to their counterparts without the induction of diabetes (control groups), indicating that knockout models may reduce ICAM-1 expression to basal levels. Our findings demonstrate that TLR2 and 4 are likely to be key mediators in regulating glomerular inflammation via ICAM-1 in the kidney. Moreover, a recent study has implicated TLR4 in modulating inflammatory processes in the mesangium [44] which further supports the role of TLR4 in perpetuating diabetic vascular complications. However, our data strongly supports the role of both TLR2 and 4 in regulating vascular inflammation via ICAM-1 expression in an *in vivo* model of diabetic nephropathy.

Collectively, our data illustrates that in the short term, TLR4 may be the more pathogenic receptor in regulating HMGB1 mediated inflammation via NF-ĸB in endothelial cells when exposed to oscillations in glucose levels. However, in chronic hyperglycemia, our in vivo data suggests the involvement of both TLR2 and TLR4 in the diabetic kidney [45]. We postulate that exposure to fluctuating glucose concentrations induce HMGB1 secretion by endothelial cells which binds to transmembrane TLR2 and 4 receptors. The activation of the signalling cascade of TLR2 and 4 by HMGB1 binding ultimately results in the translocation of NF-ĸB to the nucleus. Transcription factor NF-ĸB thereafter induces the transcription of proinflammatory cytokines and cell adhesion molecules which contribute significantly to the pathophysiology of inflammation in endothelial dysfunction relevant to diabetic microangiopathy.
